# What Is Going On? The Process of Generating Questions about Emotion and Social Cognition in Bipolar Disorder and Schizophrenia with Cartoon Situations and Faces

**DOI:** 10.3390/brainsci8040068

**Published:** 2018-04-17

**Authors:** Bryan D. Fantie, Mary H. Kosmidis, Maria Giannakou, Sotiria Moza, Athanasios Karavatos, Vassilis P. Bozikas

**Affiliations:** 1 Human Neuropsychology Guerrilla Research Group, Department of Psychology, American University, Washington, DC 20016, USA; 2 Lab of Cognitive Neuroscience, School of Psychology, Aristotle University of Thessaloniki, Thessaloniki 54124, Greece; kosmidis@psy.auth.gr (M.H.K.); mariag340@gmail.com (M.G.); stmoza@hotmail.com (S.M.); 3 Hellenic Police, General Police Directorate of Thessaly, Larissa 41334, Greece; 4 1st Psychiatry Department, Aristotle University of Thessaloniki, Thessaloniki 54124, Greece; thkarav@gmail.com (A.K.); vbozikas@med.auth.gr (V.P.B.)

**Keywords:** bipolar disorder, schizophrenia, social emotion perception, methodology, hypothesis-testing, hypothesis-generating

## Abstract

Regarding the notion of putative “best” practices in social neuroscience and science in general, we contend that following established procedures has advantages, but prescriptive uniformity in methodology can obscure flaws, bias thinking, stifle creativity, and restrict exploration. Generating hypotheses is at least as important as testing hypotheses. To illustrate this process, we describe the following exploratory study. Psychiatric patients have difficulties with social functioning that affect their quality of life adversely. To investigate these impediments, we compared the performances of patients with schizophrenia and those with bipolar disorder to healthy controls on a task that involved matching photographs of facial expressions to a faceless protagonist in each of a series of drawn cartoon emotion-related situations. These scenarios involved either a single character (Nonsocial) or multiple characters (Social). The Social scenarios were also Congruent, with everyone in the cartoon displaying the same emotion, or Noncongruent (with everyone displaying a different emotion than the protagonist should). In this preliminary study, both patient groups produced lower scores than controls (*p* < 0.001), but did not perform differently from each other. All groups performed best on the social-congruent items and worst on the social-noncongruent items (*p* < 0.001). Performance varied inversely with illness duration, but not symptom severity. Complete emotional, social, cognitive, or perceptual inability is unlikely because these patient groups could still do this task. Nevertheless, the differences we saw could be meaningful functionally and clinically significant and deserve further exploration. Therefore, we stress the need to continue developing novel, alternative ways to explore social cognition in patients with psychiatric disorders and to clarify which elements of the multidimensional process contribute to difficulties in daily functioning.

## 1. Introduction

### 1.1. Best Practices?

“There must be no barriers for freedom of inquiry. There is no place for dogma in science. The scientist is free, and must be free to ask any question, to doubt any assertion, to seek for any evidence, to correct any errors.”

[1]

“Take chances, make mistakes, and get messy.”

[2]

Recently, the notion of Best Practices has become a popular mantra for administrators throughout the business, government, higher education, and other sectors of enterprise. Best Practices are meant to represent the optimum way of doing things so that effectiveness, efficiency, and success are maximized, while simultaneously minimizing negative outcomes or even eliminating them altogether. Similar to standards of care in medicine, and purportedly established by research and evidence, Best Practices are meant to be a set of particular techniques and methodologies that all of those working in a particular field should employ. 

A complete review of all the ongoing arguments for and against the implementation of Best Practices is well beyond the scope of this paper. Nevertheless, the question before us now is whether Best Practices are compatible with the goals of science, in general, and, more importantly for the purposes of this special issue, if Best Practices are more likely to help or hinder a field with the current distinctive challenges of social neuroscience. 

It requires only commonsense when starting a new research programme to recognize the many advantages that are associated with not having to start from scratch and ‘reinvent the wheel’. Major pluses of adopting established procedures include the saving of time, effort, and cost. It is also a good idea to see how other people have done or are doing the things that one wants to do, particularly if they have done good work (and one can also benefit from seeing what has not worked as well). Learning from experience does not always need to rely on one’s own experience. After all, science is a cumulative endeavor. 

Furthermore, some level of standardization is a particularly useful practice when seeking to compare results across multiple studies. One way to enable this type of evaluation is for independent researchers to adopt common measures for investigations that share a particular aspect of the domain of interest. For example, different laboratories could use the same stimuli such as the NimStim Faces [3], the International Affective Picture System (IAPS) [4], or the OxVoc Emotional Sounds [5]. This practice can be especially effective when coupled with a set of measures like those of the NIH Toolbox [6], which make it possible to match populations sufficiently to be able to combine the results of multiple studies into a single database. 

Unfortunately, there are several troubling aspects regarding the concept of Best Practices. First, to be truly “Best” practices, the procedures must have earned this designation through research that compared all other ways of doing the things that need doing. Furthermore, if these practices are recommended for all in a field, there needs to be evidence that the practices in question are, indeed, the best for the full gamut of settings of all who will use them. The truth is, the full complement of studies needed to produce this information and support the claim of ‘Best’ have rarely been done and ‘Best’ often means ‘Best’ for us, ‘Best’ for here, and ‘Best’ for now. 

It is not unreasonable, however, to expect that, at the very least, ‘Better’ Practices can be established for many endeavors that by their explicit and concrete nature are conducive to a high level of uniformity. These activities include accounting, building a house foundation in sandy soil, operating a fast-food restaurant, treating a cardiac arrest in an ER, or running a Western blot. Nevertheless, the world is not static. Things change, there are new discoveries every day, and all practices will need eventually to adjust and adapt to external transformations in order to improve. The notion of Best Practices, however, seems to deny and resist change, which is anathema to science.

Still in its infancy, social neuroscience comprises a multiplicity of research settings, in which a wide variety of types of questions are being asked. The subject list is broad and long. Most importantly, the constructs being investigated are still evolving rapidly. There is a need for continued innovation of techniques, as well as thought, that will drive those re-conceptualizations of the major paradigms and the re-framing of the questions that will need to be answered. Change is especially relevant due to the newness of the field and because of the inherent abstract nature of the phenomena that social neuroscientists explore. 

It seems implausible to have a set of much more than largely temporary Better Practices for anything other than particular techniques, but even then, the reality of how quickly technology, information, and ideas change might limit the value of this concept. The need to find a new and better way to do things is a mainstay of science. A concept such as Best Practices is worrisome because such language has the potential to produce unnecessary and harmful constraints on the thinking and actions of researchers. It seems that State of the Art (or Science) would be a much more appropriate and positive construct. 

A discussion of Best Practices needs to include a clear description of the intended goals, outcomes, and scope of the approved procedures. As the range of prescriptive practices increases, the greater the chance of inappropriate overreach and mission drift. To be clear, some degree of standardization is a good thing and is almost always necessary, but when uniformity becomes mandated for all situations without justification, the procedures often become ritual, not scientific practice. 

Problems could arise if every study in a particular research area was done exactly the same way, with exactly the same equipment, stimuli, and procedures. It is possible in this scenario that a common, confounding influence, related directly to some flaw or feature of the shared methodological characteristics might remain undetected. Thus, even though results would replicate across independent experiments by independent laboratories, the manifesting effects would not reflect the actual relationship of the independent and dependent variables. Moreover, the results of these studies would not necessarily generalize beyond the laboratories and research and populations involved in the actual studies. 

Social neuroscience is in a developmental stage, in which there are a surfeit of hypothetical constructs that are still fluid in meaning and often employed idiosyncratically by different researchers and theoreticians. Among the primary goals in this field at this time are the discovery and determination of which hypothetical concepts are meaningful, including those not yet in the vocabulary of the field, and perhaps, not yet even recognized or conceptualized. Another goal is to determine how to operationalize these constructs appropriately and effectively. 

It is extremely unlikely that these questions will be answered in a single study, or even a dozen studies. Furthermore, the type of research one needs to do to accomplish these goals is almost certainly going to need to diverge in some ways from the classic model of hypothesis-testing, common in fields like psychology, behavioral and cognitive neuroscience, and social neuroscience. Specifically, these studies will place more of an emphasis on hypothesis-generation through exploration and discovery than on testing and confirmation. The following preliminary report describes such a study. We present it here to demonstrate how a less rigid application of the traditional procedural norms that are associated with standard hypothesis-testing, is not only appropriate, but can produce important information that can inspire and direct future investigations. 

### 1.2. The Challenge of Studying Social Neuroscience

Human beings are a social species and emotions are a fundamental part of determining almost everything we do. To function fully and effectively, we must respond appropriately to our own emotions, as well as the emotions of other individuals and groups. We respond to interpersonal stimuli, such as emotional facial expressions, prosody, and body posture. We also react to the behavior of others, incorporating what they say and what they do as individuals or as a group. Almost always, our behavior is influenced heavily by the current situation. That is, our perceptions of what is happening, where it is happening, to whom it is happening, and why it is happening, together with what has happened in the past and what we hope will happen in the future, all can contribute to how we respond or if we respond at all. 

Our reactions to these sources of input are often automatic and unconscious but they can also be learned, planned, and even calculated. When they are not automatic or not too quick to have involved significant thought, the actions we decide to take are a result of the collective influences of all that is going on and what we deem will produce the outcome that we perceive as necessary or desirable. What we can actually do is constrained by our physical abilities and the available options. If the selected response consists of multiple actions, successful completion will require that feedback be received and interpreted. This will also involve the ability to adapt behavior accordingly in real time. 

As is abundantly clear, in order to try to elucidate the phenomena related to social behavior, there are many moving parts social neuroscientists need to tease apart. We will accomplish this largely though reverse engineering and other established behavioral, cognitive, and emotional research tools and techniques as well as those that are new and novel. Deciphering the corresponding neural mechanisms and substrata is no less complicated and challenging. The traditional reductive scientific approach is to try to break complex phenomena into smaller, meaningful, functional components with the expectation that they will be easier to understand at this more basic level. The assumption is that understanding the nature of the components will aid in the development of understanding how the pieces work together to create more complicated mechanisms that can manifest more complex phenomena.

A special challenge for those who do research on emotion and other psychological phenomena is that most of the theoretical constructs that guide these studies and constrain current thinking were developed by philosophers and scientists whose ideas were formed without the benefit of what we know currently about brains, cognition, and many other sources of information relevant to understanding how these things may work. Therefore, it is vital that we remain cognizant of the fact that we frame our research questions based upon assumptions that might not actually be correct and that we need to interpret our results with enough of an open mind that it allows us to consider new and novel explanations that might not fit the current models. The truth is, we might, in fact, be thinking about emotions in completely the wrong way (see, for example, Barrett [7]. Therefore, we need to be careful not to engage in circular thinking and try to avoid falling victim to confirmation bias.

### 1.3. Dissecting the Pertinent Components

As stated earlier, effective social interaction is, for most people, an essential component of a full and satisfying life. What might seem so basic and natural to the vast majority of us is actually much more complex. Successful interpersonal and group behavior requires an individual to be able to perceive and act on a panoply of signs and stimuli. At the molar level, this includes almost simultaneously perceiving the pertinent social and situational contexts, interpreting one’s own emotion as well as that of others, and being able to produce the appropriate emotional and behavioral responses that will lead to an effective and desirable outcome. A failure at any level of these processes can produce problems that are able to impair everyday functioning. The more complex a behavior is, the more vulnerable it is to disruption by the malfunction of any of its components. Thus, it is paramount to identify the weak link or links that result in compromised social behavior. 

One approach to address this issue is to gather information about the functional integrity of the complex behavior as a whole, such as when it occurs in a natural setting, following that with an assessment of how well each of the dissociable component behavioral elements is working separately. In the case of social behavior, these components can include input-related processes, such as the ability to identify facial expressions or tone of voice, as well as behavioral output-related processes, such as response selection, response inhibition, or the ability to modify on-going responses. Furthermore, because of the additive and integrative nature of the components, it would be useful to test how multiple configurations of these functional elements work together. The eventual aim, therefore, would be to have a full profile of performances on tasks that have a range of demands, starting as simple as the perception of a set of specific stimuli to the complexity of real-life situations with as many meaningful intermediate steps as are necessary to produce a coherent model of this behavior.

More specifically, the plan of attack would start with the use of broad behavioral measures to identify a population that appeared to be demonstrating impaired social behavior. With that established, follow-up studies could be designed to examine how well members of the affected group do on a series of assessments that focus on increasingly simplified versions of the behavioral (e.g., actions) and informational (e.g., relevant stimuli) components involved in real-life settings. For example, these tasks could range in complexity from having the research participants watch and interpret the actions of real people interacting on videos to having them identify the emotions expressed in photographs of people’s faces in which only the eyes are visible. 

One possible intermediate step in this sequence could be to have people identify the facial expression that they consider appropriate for a cartoon depicting an emotion-provoking situation. This task, which we will discuss subsequently in more detail, would involve only a subset of the abilities typically required for effective social interaction. The cartoon situations would be much less complex and contain fewer cues, and distractions, than either video or live, dynamic action by real-life characters or actors. If a group known to have problems with social interaction could do as well as controls did in identifying the cartoon situations and matching them to the appropriate facial expressions, one might then be encouraged to seek more complex aspects of the behavior to assess in order to find the source of the difficulty. On the other hand, if a group has trouble with the cartoon-face matching task, it might be best to examine the more basic perceptual or response-related components that go into performing the task successfully for clues to what the impairment might be. 

### 1.4. Studying Patient Populations

There are two patient populations, comprising those with either a diagnosis of schizophrenia or bipolar disorder, about whom we have a particular scientific and clinical interest, and for whom a better understanding of the processes and mechanisms involved in interpersonal behavior might prove beneficial. Previous studies have demonstrated impaired social and interpersonal behavior in both groups. Significantly, rich social networks are associated with greater subjective feelings of well-being, as well as better brain health and recovery from or adjustment to psychiatric disorders and trauma [8]. Therefore, better comprehension of the dysfunction in the socio-emotional domain associated with these disorders, and others, is likely to generate greater insight into the nature of the challenges faced by those affected and support the development of more effective methods of diagnosis and treatment.

An additional area of interest was the comparison of patients with bipolar disorder and those with schizophrenia as, perhaps, a means to help elucidate further the pathophysiological mechanisms that might underlie difficulties in social emotion perception. Given the apparent overlap, both phenotypic and genotypic, of these disorders [9], but differences often reported in their functional outcome during remission [10], a direct comparison might shed some light on the putative presence of a continuous psychotic spectrum. Therefore, patients with schizophrenia would appear to be an ideal psychiatric comparison group for patients with bipolar disorder I (BD).

Comparing and contrasting two disorders, like schizophrenia and bipolar disorder with overlapping behavioral, cognitive, and emotional symptoms, overlapping disorder-related neural substrata, and overlapping genetic profiles is a fundamental research paradigm in neuropsychological and social neuroscience. These relationships allow one to compare and contrast differences and similarities between the two disorders, focused on identifying what brain systems or genetic profiles correspond with the presence or absence of each set of behavioral, cognitive, and emotional symptoms. The essential principle is to correlate what features of each of these dimensions (i.e., genes, brain systems, behavior-cognition-emotion) are shared between these disorders and what features are separate and distinct to each.

### 1.5. Previous Research

Previous research has focused primarily on the use of similar tasks to explore emotion perception in psychiatric patients. Most have relied on photographs of faces showing a particular emotion [11,12,13,14,15,16,17,18,19,20,21,22,23,24,25,26,27], requiring the examinee to identify them by choosing from a selection of labels of emotions or to match two stimuli depicting the same emotion. While most of these used staged emotions expressed in static photographs for the purpose of the development of the particular tasks, others used naturalistic emotional expressions (i.e., photographs of real life facial expressions from magazines [28]). In all cases, the faces were presented alone, without contextual information. Several investigations have used prosodic information indicating particular emotions [17,24,29,30,31,32,33], also as stand-alone stimuli, and not within a social or other context. Fewer studies have used videotaped expressions of emotions in faces [12,34,35,36,37], again limited to the stimuli of interest with no background or context.

With few exceptions, the aforementioned studies found some form of impairment in patients with schizophrenia, and, less often, in those with bipolar disorder, in emotion perception relative to healthy comparison groups. While a detailed review is beyond the scope of the present investigation, these impairments are important clinically as they have been linked to below par social functioning, interpersonal problem solving [38], and quality of life [24]. Some ambiguity still exists regarding the ubiquitousness of this emotion perception impairment in patients with bipolar disorder, because in some studies they performed better than patients with schizophrenia [25], or even as well as their healthy peers [11]. Perhaps this reflects differences in patient characteristics, particularly with respect to stage of disease, duration of illness, etc., methodological differences across studies, or both.

### 1.6. Matching Photos of Emotional Facial Expressions to Situations Depicted in Cartoons

The Fantie Cartoon Task [39] we are using in the current study is an elaboration and expansion of a task created originally by Kolb and Whishaw [28]. Kolb and Whishaw used their cartoon task in a mini-battery of facial affect perception tasks. These included a task in which the participant was asked to name the emotions expressed by faces taken from photographs that appeared in Life Magazine, featuring people in real-life situations, as well as other faces from Ekman’s classic stimuli and line drawings of faces expressing emotions [40]. The participants then had to match each of these photo faces to one of a set of six key photo faces, each of which expressed one of Ekman’s original six basic emotions (Note: The stimuli in all these tasks involved only the faces). 

The cartoon situation task from which the current measure evolved required participants to match one of six key emotional faces to a character in each of the cartoon situations whose face was left blank. When they administered this and the other tasks in the mini-affect battery to a group of people with schizophrenia, Kolb and Whishaw [28] found that the patients could name and match the faces by emotion as well as the healthy controls and those in a Tourette’s group, which they included to control for the effects of the anti-dopaminergic medications both patient groups had been prescribed. They reported that those with schizophrenia, however, did not do as well on the cartoon task as the other two groups. Kolb and Whishaw attributed this to a possible difficulty in identifying the social context provided by the cartoon.

As well as a larger set of cartoon situations, and a neutral expression answer option, the current version includes the systematic inclusion of social and non-social items in addition to social items that differ in congruency between the emotion the faceless protagonist should be showing and that displayed by the other characters in the cartoon situations. These additions should allow us to explore two more conditions that might impede or enhance the performance of our two patient groups. It is important to note that we are not looking merely for evidence of impaired performance, which might be the result of a general performance deficit related to the disorders or the medications people might be taking and might not be specific to recognizing emotions. We are also looking for patterns of relative strengths and weaknesses that might differentiate the patient groups and provide some additional insight about the processes involved in these differences, if they exist. 

### 1.7. Aim and Hypotheses of the Present Study

In summary, our goal was to eliminate some of the potential variables involved in the decoding and understanding of complex live-action or video-taped emotion-provoking scenarios. In this way, the demands of the task would be limited more to the interpretation of the simplified situational cues available in the cartoons and selecting the appropriate emotional expression from an array of still photographs of faces. Therefore, we created cartoons of everyday scenarios depicting both social (i.e., involving more than one person) and nonsocial (i.e., involving only one person) events. In each of these cartoons, the face of one character, to whom we will refer as the ‘protagonist’, was left blank. The task required the participant to choose the appropriate expression, which the blank face should be making in that situation, from an array of photographs of standardized emotional expressions.

The social situations contained another dimension—the relationship between what the protagonist’s emotional expression should likely be in the depicted situation and that of the facial expressions of the others in the scene. In the Congruent condition, the protagonist’s expected expression should be the same as that of everyone else in the scene (e.g., everyone depicted is on a rollercoaster). In the Noncongruent condition, one should expect that the protagonist would display an emotional expression different from that of the other people in the scene (e.g., the protagonist has just set off a firecracker that has startled a group of playmates). 

Through this approach, we hoped to assess the ability of our study’s participants to use contextual cues of a social nature to infer what the absent emotional expression of the protagonist should be, as appropriate to each depicted situation. Would we observe a dissociation between the processes of matching faces to nonsocial cartoons and that of matching faces to social situations? Would it make a difference, if, in the social cartoons, the emotions of the people depicted were the same or conflicting? We also sought to explore potential differences among particular emotions as they relate to contextual cues; would any failure be limited to certain emotions or would it be generalized? Finally, we explored the potential relationship between patients’ performance on these tasks and their clinical symptoms. 

## 2. Materials and Methods

### 2.1. Participants 

Participants belonged to one of three groups: 19 (8 men) patients with bipolar disorder I (BD) in remission, 32 (18 men) patients with schizophrenia (SCH), and 36 (22 men) healthy controls (HC). We recruited the patients with bipolar disorder and those with schizophrenia from the outpatient service of two university psychiatric clinics and the healthy controls from the community. All participants gave their informed consent for inclusion before they participated in the study. The study was conducted in accordance with the Declaration of Helsinki [41]).

The three groups did not differ significantly in terms of age (F(2,84) = 0.891, *p* = 0.414), and level of education (F(2,84) = 1.814, *p* = 0.169). Also, the two patient groups did not differ with respect to duration of illness (t(49) = 0.906, *p* = 0.369). 

All patients were diagnosed according to DSM-IV criteria [42]. Diagnosis was confirmed with the Greek version (translation-adaptation to the Greek language by S. Beratis) of the Mini International Neuropsychiatric Interview (4.4) (MINI) [43]. Symptom severity of patients with BD was assessed by the Montgomery–Asberg Depression Rating Scale (MADRS) [44] and the Young Mania Rating Scale (YMRS) [45]. We used a cut-off score of 10 on each of these scales as our criterion of remission. Symptom severity (positive symptoms, negative symptoms, and general psychopathology) of patients with SCH was assessed with the Greek version [46] of the Positive and Negative Syndrome Scale (PANSS) [47]. All patients with schizophrenia had either a score of less than three on each item of the positive subscale of the PANSS or had exhibited no changes in their positive subscale scores for at least two months prior to participation in the study and, therefore, their condition was considered stable. These scales were administered by a psychiatrist (VPB). Patients with schizoaffective disorder were excluded. Demographic characteristics of the three groups, as well as patient group clinical characteristics are presented in Table 1. 

All patients with BD and SCH were receiving medication at the time of the experiment: patients with BD, anti-epileptics, atypical antipsychotics, and antidepressants, either as monotherapy or in various combinations, and patients with SCH, mainly atypical antipsychotics.

Exclusion criteria for patient groups included a history of neurological and developmental disorders, head injury with loss of consciousness, alcohol or drug abuse during the six-month period prior to testing, and any physical illness that may have affected cognitive performance. Additional criteria for excluding healthy participants were a history of any psychiatric disorder or treatment, as well as a family history of depression, psychosis, or bipolar disorder. One of the authors (Maria Giannakou) interviewed all healthy participants before they entered the study.

### 2.2. Procedure

We administered a computerized test consisting of 57 drawings (or cartoons), each one depicting an everyday scenario with one or more people, wherein the face of the protagonist was missing [21,39]. On each trial, a series of seven photographs of Ekman faces depicting the classic basic emotional expressions (i.e., happiness, sadness, surprise, anger, fear, and disgust), as well as a neutral expression [40] appeared at the bottom of the computer screen below the cartoon drawing. Instructions were as follows: “Please select the facial expression among these photographs that matches best what this person without a face must be feeling. Make your selection regardless of the gender or age of the person in the photograph”. Thus, participants needed to decode and interpret the social scenario in order to select the one of the seven emotional expressions that fit the missing face best.

Each of the 57 stimuli matched one of three conditions, according to the context of the cartoon situation. These included: (1) “nonsocial” (depicting only one person; 19 stimuli); (2) “social congruent” (with more than one person, wherein the expression of the missing face should match that of the others in the scene; 22 stimuli); and (3) “social noncongruent” (wherein the expression of the missing face should be different from those of the others in the scene; 16 stimuli). 

**Figure 1:**
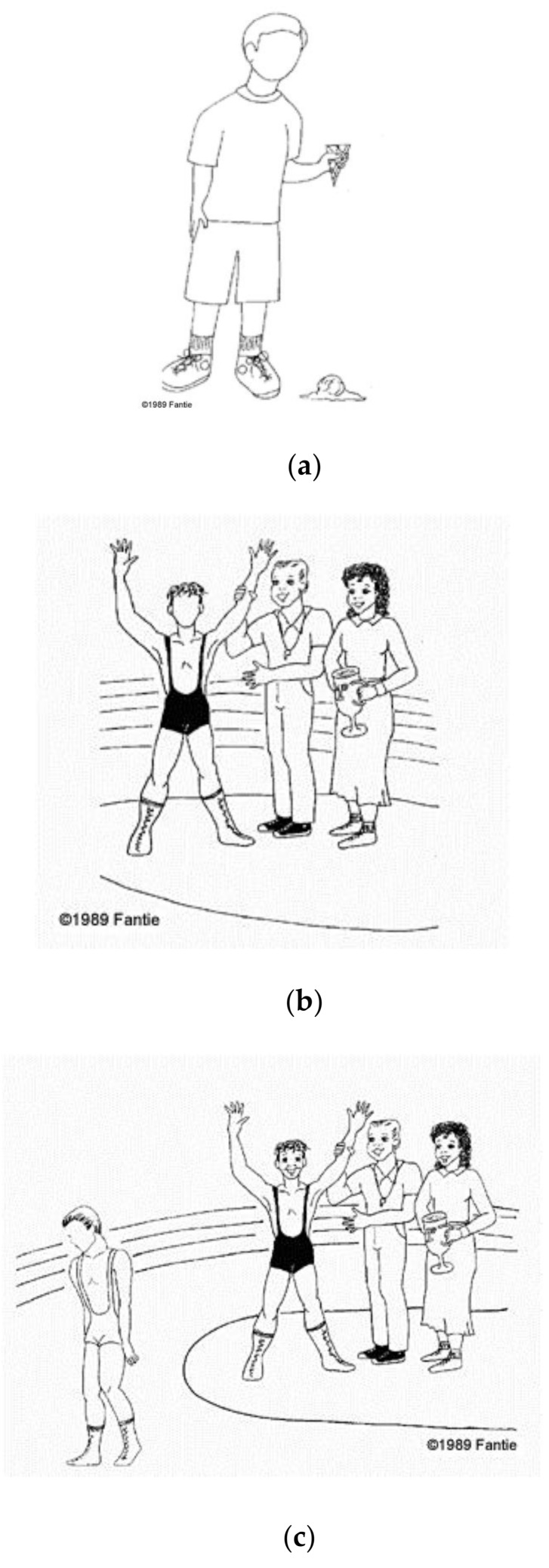
Examples of test items: (**a**) nonsocial context item; (**b**) social congruent context item; (**c**) social noncongruent context item.

An example of a “nonsocial context” is a cartoon in which the protagonist with a missing face is a lone child looking at the scoop of ice cream that had just fallen onto the ground from the cone he is holding (Figure 1a). An example of a “social congruent context” stimulus is a cartoon in which the protagonist with a missing face is a wrestler who, presumably, is happy to have won a match surrounded by smiling officials giving him his trophy (Figure 1b); an example of a “social noncongruent context” stimulus is a cartoon in which the protagonist with a missing face is a wrestler who, presumably, is sad because he has lost the match, with the winner and officials smiling in the background (See Figure 1c). 

## 3. Results

### 3.1. Scoring Procedure, Variables, and Statistical Analyses

Because some items could have two or more reasonable answers (e.g., a surprise birthday party could induce feelings of surprise or happiness), we did not use the response that was intended originally by the designer of the task (BDF) to determine a priori a single response choice for each cartoon that we would consider as the ‘correct’ answer for each item. Instead, we scored responses for each scenario based on the proportion of the healthy control group who selected each possible answer. For example, on item 14 (depicting two people, one of whom is about to drink punch from a punchbowl in which there is a frog), 80% of the healthy control participants responded ‘disgust’, 6% ‘surprise’, 5% ‘anger’, 4% ‘fear’ and 2% for each of the other emotions (i.e., ‘happiness’, ‘sadness’, and ‘neutral’, respectively). Therefore, any patient or healthy control who responded ‘disgust’ received a score of 80 for this response, those who responded ‘surprise’ received a 6, ‘anger’ a 5 and so forth. Thus, each possible response option for each cartoon generated a score. The value of that score, which can range from 0 (not unusual) to 100 (almost never), is based on the proportion of the Control group who chose that option. These proportions can and do differ among individual items and this is reflected in the weightings. 

We calculated the weightings in order to capture the reality that not all items are equal. That is, some scenarios produce just one or two responses from the healthy controls (e.g., the person entering the room to find a waiting surprise party is typically matched with the Surprised or Happy facial expression and rarely anything else). Other scenarios can result in the members of the control group splitting into roughly equal numbers of people choosing one of three or four facial expressions (e.g., the person stepping in the dog feces can elicit matches with faces that are disgusted, angry, sad, or neutral). When the consensus of the controls is high for a particular emotion-scenario pairing, such as when everyone in the control group selects just one or two expressions for a particular cartoon, those items have a larger impact on the total score of the individual participant than items for which the consensus is weaker. Along the same lines, if an individual selected a response option for a cartoon that no one else selected, that “incorrect” response should have a greater impact on the final score for that individual when the item was one for which there was high consensus for only one or two responses among controls compared to items in which the control group was split among several responses. The more controls agree on which emotional expressions should match a particular cartoon, the more selecting that response choice adds to the individual’s score. The more aberrant the choice, the more of a negative impact it has on the score. 

Theoretically, the range of mean scores should be 0–100%, but this has never happened in our experience with this task because there is rarely, if ever, 100% agreement of a group of healthy controls for any individual cartoon scenario, let alone for 57 scenarios. We have analyzed data with the scores presented as the raw mean weighted score or the score represented as the percentage of the highest maximum score possible based on the mean of the highest weighted responses for all 57 items, and, not surprisingly the statistical analysis came out exactly the same. 

We used repeated measures analyses of variance to compare the three groups on the total mean score on each of the three conditions, yielding a 3 (group) × 3 (condition) analysis, as well as to compare the three groups on two dimensions, yielding a 3 (group) × 2 (social–nonsocial context) and a separate 3 (group) × 2 (congruent–noncongruent social context) analysis. We also compared the groups on their mean weighted scores by emotion in a group (3) × emotion (6) analysis.

### 3.2. Group × Condition

**Figure 2:**
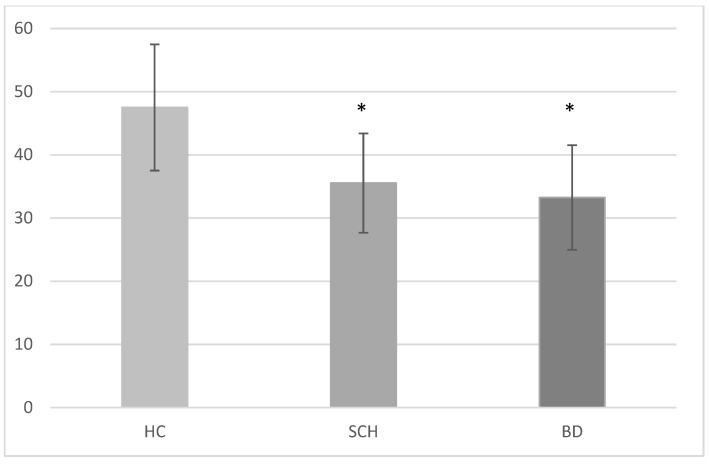
Mean score on total task by group. * Healthy control group > schizophrenia group = bipolar group.

**Figure 3:**
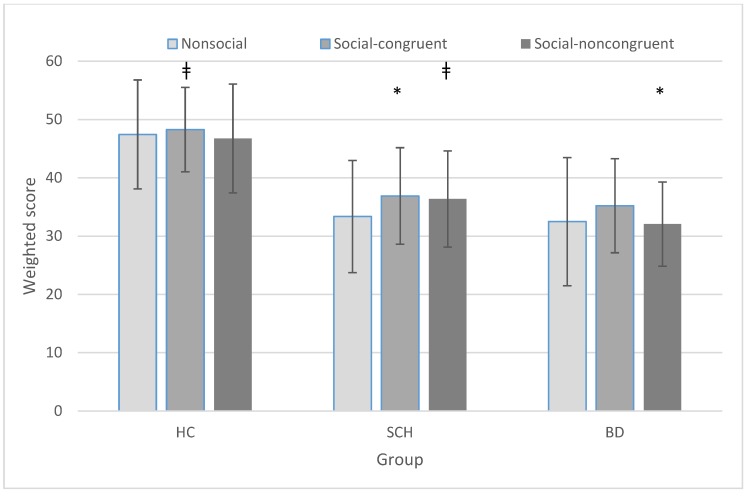
Mean score on each test condition by group. BD = bipolar disorder group; SCH = schizophrenia group; HC = healthy control group ǂ Social-congruent > nonsocial = social-noncongruent; * Healthy control group > schizophrenia group = bipolar group.

Figure 2 and Figure 3 reflect the group × condition comparison. We found a group main effect (F(2,84) = 25.850, *p* < 0.001, η^2^ = 0.381, observed power = 1.000), wherein the choices of expressions made by both patient groups differed from those made by the healthy control group, and they did not differ from each other. We also found a condition main effect (F(2,168) = 7.119, *p* = 0.001, η^2^ = 0.078, observed power = 0.928), indicating greater consensus concerning the choice of emotion on the social-congruent scenarios than either of the two others, but a marginal group × condition interaction (F(4,168) = 2.358, *p* = 0.056, η^2^ = 0.053, observed power = 0.673). 

When comparing the groups on nonsocial and social conditions (the latter including both congruent and noncongruent social contexts), we found a group (F(2,84) = 27.504, *p* < 0.001, η^2^ = 0.396, observed power = 1.000) and a condition main effect (F(1,84) = 7.816, *p* < 0.001, η^2^ = 0.085, observed power = 0.789), as well as a group × condition interaction (F(2,84) = 3.826, *p* = 0.026, η^2^ = 0.396, observed power = 0.680). Similarly, when comparing the groups only on the two types of social items (congruent vs. noncongruent context), we found both a group (F(,2,84) = 21.604, *p* < 0.001, η^2^ = 0.340, observed power = 1.000) and a condition main effect (F(1,84) = 6.538, *p* = 0.012, η^2^ = 0.072, observed power = 0.715), but no interaction (F(2,84) = 1.135, *p* = 0.326, η^2^ = 0.026, observed power = 0.244). 

### 3.3. Group × Emotion 

**Figure 4:**
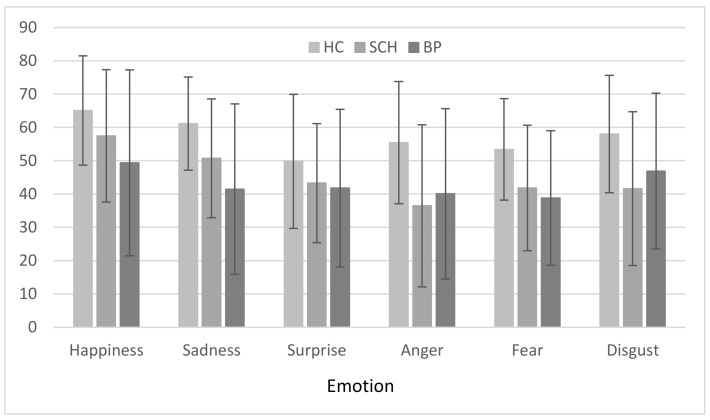
Mean scores on each emotion for each group.

Figure 4 depicts the group x emotion comparisons. A repeated-measures ANOVA indicated a group effect overall (F(2,84) = 7.505, *p* = 0.001, η^2^ = 0.152, observed power = 0.936); Bonferroni post hoc comparisons indicated that the choices of expressions by patient groups deviated from those of the healthy control group (both *p*’s = 0.005), but did not differ from each other (*p* = 1.00). We also found an emotion main effect (F(5,420) = 10.222, *p* < 0.001, η^2^ = 0.108, observed power = 1.000). Post hoc comparisons of emotions to each other yielded higher scores on items indicating happiness over all other emotions (*p*’s ranging from <0.001–0.011), and higher scores on items indicating sadness than on those indicating anger (*p* = 0.026) and fear but not surprise (*p* = 0.037). There was no group × emotion interaction (F(10,420) = 1.660, *p* = 0.088, η^2^ = 0.038, observed power = 0.800).

### 3.4. Correlations

We conducted Pearson correlations for the two patient groups. Specifically, we explored potential associations between duration of illness (in years) and measures of symptom severity (MADRS and YMRS total scores for the BD group and PANSS general psychopathology for the schizophrenia group) on the one hand and performance on each of the three conditions of the task and on each of the six emotions. We found significant negative correlations between duration of illness (for both groups together) and nonsocial scenarios (r(49) = −0.369, *p* = 0.005), fear (r(49) = −0.401, *p* = 0.002) and disgust (r(49) =−0.247, *p* = 0.044); the longer the illness, the lower the scores on these measures. Additionally, our results indicated a positive correlation between the MADRS and the nonsocial (r(17) = 0.507, *p* = 0.019) and social (r(17) = 0.433, *p* = 0.041) conditions, as well as with sadness (r(17) = 0.497, *p* = 0.022) and surprise (r(17) = 0.465, *p* = 0.030). General psychopathology PANSS scores and YMRS scores did not correlate with any task variable.

## 4. Discussion

The present findings revealed that both patient groups produced lower scores than their healthy peers on this task of matching photos of key emotional facial expressions to cartoons of everyday scenarios. This pattern of lower scores on a task involving faces and emotion by the patients with schizophrenia is consistent with other similar studies [17,20,21,26]. It would be tempting to claim that the difference between the level of performance of the patient groups compared to the healthy controls was the result of the patient groups’ impairment in understanding the scenarios, recognizing the emotions expressed by the faces, decoding social inference, or knowing what the correct emotional responses should be. To do so, however, would be to fall victim to seductive inference. Just because successful performance on this task should depend on the ability to assess social inference, for example, or decode social situations, does not mean that this particular component of the process is impaired. In fact, there is more than one way that complicated problems can be solved that are not always apparent (e.g., Clever Hans [48]). Conversely, there are even more ways that performance can be affected negatively in addition to an impairment of the particular ability targeted by the investigators. Before one can claim specific explanations for an observed effect, one must eliminate simpler causes, such as a general performance effect or differing levels of effort or attention between groups. This is extremely difficult to do when using only a single measure in a study.

The performance of all three groups was best on the social-congruent items, and all three groups did worse on the social-noncongruent scenarios. Thus, all three groups shared the same performance profile across the three different scenario context conditions. In the social-congruent condition, people in the cartoon, other than the faceless protagonist, express what is the appropriate choice of facial expression for everyone in that situation. It is possible that their expressions could have served as a hint to the “right” answer, even if one or more of the other response choices might have seemed acceptable. Another possibility is that situations in which everyone is expressing the same emotion are less complex and easier to interpret. In addition, the presence of the congruent faces might have increased the level of consensus for the most popular response and this would also produce higher scores for the items in this category due to the weighting procedure. Of course, more than one of these explanations might be correct in addition to others not yet known.

Similarly, in the social-noncongruent items, the faces of people other than that of the protagonist might have acted as distractors and increased the ambiguity of the situations, thus leading more participants to more choices of less popular answers thus reducing the degree of consensus associated with the responses, which would also lower the weighted scores. Notably, the differences between performances on the three types of scenario contexts were small (i.e., >10%). Furthermore, the differences between the overall performances of the two patient groups compared to the healthy controls was roughly 25%. In addition, those who had trouble understanding the cartoon situations or who were not making their best efforts, might have chosen to select the facial expression that resembled the faces they could see in the cartoon. Anyone who used that strategy would get most of the Congruent items correct and most of the Noncongruent items wrong, which would have produced much larger differences between the performance of these two conditions than what we observed. 

In light of these findings, it seems clear that the patient groups in the current study were capable of doing this task and one might safely be able to rule out any sort of complete emotional, social, cognitive, or perceptual inability. Nevertheless, the differences in performance between patients and healthy controls could be meaningful functionally and clinically significant. Even small errors, miscues, or misunderstandings can have a large negative impact on social and interpersonal behavior. 

It might be worth noting that the patients with bipolar disorder in remission showed a level of performance that was lower than that of the patients with schizophrenia across all task conditions, although this was not significant statistically in this preliminary study. It is more usual for patients with schizophrenia to do worse than other patient groups on most tests of this kind because of the pervasive nature of the disorder and its detrimental effects upon multiple cognitive and emotional domains. 

By comparing the two patient groups to each other based on their performance in toto, as well as on specific emotions (that is, their concurrence with the emotion with the highest weighted score on each scenario), we had hoped to shed some light on the question of whether these disorders could be seen as occurring on a psychosis-related continuum and whether their differences were qualitative or merely quantitative. Had we found better performance of the BD group relative to the schizophrenia group, we would have surmised that emotion perception in social scenarios was influenced by level of pathology. 

Additionally, had we detected a distinctive pattern of performance in each group relative to the individual emotions, it may have helped to elucidate the underlying reasons for these differences in each pathological condition. Yet, our findings do not support such a differentiation. Also, with respect to the lower scores on the negative emotion-related items, similar results in other studies using other measures, have produced potential explanations based on the notion of the increased evolutionary pressure to detect social signals of negative valence as potential warnings of potential danger; an alternative explanation, however, is that it may be an artifact of stimulus difficulty levels [49,50]. 

Alternatively, the difference between performance on negative and positive emotion-related items might simply be because there is only one truly positive emotion in the response options, that is, ‘Happy’. To make the correct response for positive items, one need only recognize the valence of the situation to match it to the one positive face in the response choices. In contrast, with negatively-valenced items there are at least four negative options and to respond correctly one must be able to differentiate between individual emotions. In any case, the patterns of difficulties that we observed in the two patient groups in the present study did not diverge in a meaningful manner.

The purpose of the study we have described was exploratory and the goal was not to create a standardized measure of a hypothetical construct, nor are we suggesting that this task be used as a clinical diagnostic instrument. Rather, we were using this task as a behavioral assay, which has the potential to produce a rich variety of behavioral data that can be used in subsequent studies, in conjunction with other sources of data, to generate and support hypotheses about emotional processes in the examined patient groups as well as in healthy controls. 

## 5. Conclusions

This preliminary research report represents hypothesis-generating research, not hypothesis testing. All we are asking the reader to conclude at this time is that there might be something interesting going on that deserves further exploration; moreover, we wished to introduce the reader to a novel tool that might help inspire innovative ways of asking new questions. Our findings also do suggest that it might be best for follow-up studies directed at the same questions to employ tasks that are either the same or less complex than those used in the current study. If there is a measurable difference between the performances of these patient groups and healthy controls on this cartoon task, it would be highly unlikely (but not impossible) for there not to be at least equal differences on more complex and demanding tasks. Also based on the current results, the first important step would be to see if we could rule out some nonspecific, general performance effect or lack of effort as the source of the discrepancy between performances. If we can exclude those explanations, the root problems are apt to be found in the dysfunction of more basic processes. 

At this stage of the exploration, the precise nature of what is going on at the behavioral, cognitive, and affective levels, what neural systems are involved, as well as which processes are affected and how, are for studies yet to come to investigate and confirm. Based on this approach, we stress the need for developing alternative ways to explore social context interpretation in patients with a psychiatric disorder to elucidate further the elements of the process that contribute to their difficulties in daily functioning.

## Figures and Tables

**Table 1:** Demographic characteristics of all participants and clinical characteristics of the patient groups.

	BD		SCH		HC	
	*M* (*SD*)	Range	*M* (*SD*)	Range	*M* (*SD*)	Range
Age (years)	39.32 (10.72)	24–57	37.00 (9.84)	21–57	35.55 (9.61)	21–61
Education (years)	12.16 (2.83)	6–18	10.72 (3.19)	6–16	11.55 (2.10)	6–16
Duration of illness (years)	13.16 (10.24)	2–36	10.85 (8.51)	0.5–36	-	-
MADRS	1.53 (2.61)	0–8	-	-	-	-
YMRS	3.16 (2.48)	0–8	-	-	-	-
PANSS						
Positive symptoms	--	--	14.66 (8.49)	7–30	-	-
Negative symptoms	--	--	20.38 (6.10)	10–35	-	-
General psychopathology	--	--	27.56 (5.90)	17–42	-	-

BD = bipolar disorder group; SCH = schizophrenia group; HC = healthy control group; M = Mean; SD = standard deviation; MADRS = Montgomery–Asberg Depression Rating Scale; YMRS = Young Mania Rating Scale; PANSS = Positive and Negative Symptom Scale.

## References

[1] Oppenheimer J.R. (1949). Life Magazine.

[2] Frizzle V.F. (1994). The Magic School Bus. https://en.wikipedia.org/wiki/The_Magic_School_Bus.

[3] Tottenham N., Tanaka J.W., Leon A.C., McCarry T., Nurse M., Hare T.A., Nelson C. (2009). The NimStim set of facial expressions: Judgments from untrained research participants. Psychiatry Res..

[4] Lang P.J., Bradley M.M., Cuthbert B.N. (2008). International Affective Picture System (IAPS): Affective Ratings of Pictures and Instruction Manual.

[5] Parsons C.E., Young K.S., Craske M.G., Stein A.L., Kringelbach M.L. (2014). Introducing the Oxford Vocal (OxVoc) Sounds database: A validated set of non-acted affective sounds from human infants, adults, and domestic animals. Front. Psychol..

[6] Weintraub S., Dikmen S.S., Heaton R.K., Tulsky D.S., Zelazo P.D., Bauer P.J., Gershon R.C. (2013). Cognition assessment using the NIH Toolbox. Neurology.

[7] Barrett L.F. (2017). The theory of constructed emotion: An active inference account of interoception and categorization. Soc. Cognit. Affect. Neurosci..

[8] Perkins J.M., Subramanian S.V., Christakis N.A. (2015). Social Networks and Health: A Systematic Review of Sociocentric Network Studies in Low- and Middle-Income Countries. Soc. Sci. Med..

[9] Clay H.B., Sillivan S., Konradi C. (2011). Mitochondrial dysfunction and pathology in bipolar disorder and schizophrenia. Int. J. Dev. Neurosci..

[10] Pacheco A., Barguil M., Contreras J., Montero P., Dassori A., Escamilla M.A., Raventós H. (2010). Social and clinical comparison between schizophrenia and bipolar disorder type I with psychosis in Costa Rica. Soc. Psychiatry Psychiatr. Epidemiol..

[11] Addington J., Addington D. (1998). Facial affect recognition and information processing in schizophrenia and bipolar disorder. Schizophr. Res..

[12] Baez S., Herrera E., Villarin L., Theil D., Gonzalez-Gadea M.L., Gomez P., Ibañez A.M. (2013). Contextual Social Cognition Impairments in Schizophrenia and Bipolar Disorder. PLoS ONE.

[13] Bozikas V.P., Tonia T., Fokas K., Karavatos A., Kosmidis M.H. (2006). Impaired emotion processing in remitted patients with bipolar disorder. J. Affect. Disord..

[14] Daros A.R., Ruocco A.C., Reilly J.L., Harris M.S.H., Sweeney J.A. (2014). Facial emotion recognition in first-episode schizophrenia and bipolar disorder with psychosis. Schizophr. Res..

[15] Edwards J., Jackson H.J., Pattison P.E. (2002). Emotion recognition via facial expression and affective prosody in schizophrenia: A methodological review. Clin. Psychol. Rev..

[16] Edwards J., Jackson H.J., Pattison P.E. (2002). Erratum to “Emotion recognition via facial expression and affective prosody in schizophrenia: A methodological review” [Clinical Psychology Review 22 (2002) 789–832]. Clin. Psychol. Rev..

[17] Edwards J., Pattison P.E., Jackson H.J., Wales R.J. (2001). Facial affect and affective prosody recognition in first-episode schizophrenia. Schizophr. Res..

[18] Goghari V.M., Sponheim S.R. (2013). More pronounced deficits in facial emotion recognition for schizophrenia than bipolar disorder. Compr. Psychiatry.

[19] Harmer C.J., Grayson L., Goodwin G.M. (2002). Enhanced recognition of disgust in bipolar illness. Biol. Psychiatry.

[20] Kohler C.G., Hoffman L.J., Eastman L.B., Healey K., Moberg P.J. (2011). Facial emotion perception in depression and bipolar disorder: A quantitative review. Psychiatry Res..

[21] Kosmidis M.H., Bozikas V.P., Giannakou M., Anezoulaki D., Fantie B.D., Karavatos A. (2007). Impaired emotion perception in schizophrenia: A differential deficit. Psychiatry Res..

[22] Lembke A., Ketter T.A. (2002). Impaired Recognition of Facial Emotion in Mania. Am. J. Psychiatry.

[23] Meehan K.B., De Panfilis C., Cain N.M., Antonucci C., Soliani A., Clarkin J.F., Sambataro F. (2017). Facial emotion recognition and borderline personality pathology. Psychiatry Res..

[24] Minzenberg M.J., Poole J.H., Vinogradov S. (2006). Social-emotion recognition in borderline personality disorder. Compr. Psychiatry.

[25] Ruocco A.C., Reilly J.L., Rubin L.H., Daros A.R., Gershon E.S., Tamminga C.A., Sweeney J.A. (2014). Emotion recognition deficits in schizophrenia-spectrum disorders and psychotic bipolar disorder: Findings from the Bipolar-Schizophrenia Network on Intermediate Phenotypes (B-SNIP) study. Schizophr. Res..

[26] Sachs G., Steger-Wuchse D., Kryspin-Exner I., Gur R.C., Katschnig H. (2004). Facial recognition deficits and cognition in schizophrenia. Schizophr. Res..

[27] Venn H.R., Gray J.M., Montagne B., Murray L.K., Burt D.M., Frigerio E., Young A.H. (2004). Perception of facial expressions of emotion in bipolar disorder. Bipolar Disord..

[28] Kolb B., Whishaw I.Q. (1982). Production and Perception of Facial Expression: A Comparison of Focal Brain Lesions and Schizophrenia.

[29] Bozikas V.P., Kosmidis M.H., Anezoulaki D., Giannakou M., Andreou C., Karavatos A. (2006). Impaired perception of affective prosody in schizophrenia. J. Neuropsychiatry Clin. Neurosci..

[30] Bozikas V.P., Kosmidis M.H., Tonia T., Andreou C., Focas K., Karavatos A. (2007). Impaired perception of affective prosody in remitted patients with bipolar disorder. J. Neuropsychiatry Clin. Neurosci..

[31] Leitman D.I., Foxe J.J., Butler P.D., Saperstein A., Revheim N., Javitt D.C. (2005). Sensory Contributions to Impaired Prosodic Processing in Schizophrenia. Biol. Psychiatry.

[32] Leitman D.I., Laukka P., Juslin P.N., Saccente E., Butler P., Javitt D.C. (2010). Getting the Cue: Sensory Contributions to Auditory Emotion Recognition Impairments in Schizophrenia. Schizophr. Bull..

[33] Leitman D.I., Wolf D.H., Laukka P., Ragland J.D., Valdez J.N., Turetsky B.I., Gur R.C. (2011). Not Pitch Perfect: Sensory Contributions to Affective Communication Impairment in Schizophrenia. Biol. Psychiatry.

[34] Bellack A.S., Blanchard J.J., Mueser K.T. (1996). Cue availability and affect perception in schizophrenia. Schizophr. Bull..

[35] Cramer P., Bowen J., Oneill M. (1992). Schizophrenics and Social Judgement: Why Do Schizophrenics Get It Wrong?. Br. J. Psychiatry.

[36] Joseph P.L.A., Sturgeon D.A., Leff J. (1992). The Perception of Emotion by Schizophrenic Patients. Br. J. Psychiatry.

[37] Kosmidis M.H., Aretouli E., Bozikas V.P., Giannakou M., Ioannidis P. (2008). Studying social cognition in patients with schizophrenia and patients with frontotemporal dementia: Theory of mind and the perception of sarcasm. Behav. Neurol..

[38] Corrigan P.W., Toomey R. (1995). Interpersonal problem-solving and information-processing in schizophrenia. Schizophr. Bull..

[39] Fantie B.D. (1995). Fantie Cartoon Task.

[40] Ekman P., Oster H. (1979). Facial Expressions of Emotion. Annu. Rev. Psychol..

[41] World Medical Association (2013). World medical association declaration of Helsinki: Ethical principles for medical research involving human subjects. JAMA.

[42] APA (1994). Diagnostic and Statistical Manual of Mental Disorders: DSM-IV.

[43] Sheehan D.V., Lecrubier Y., Sheehan K.H., Amorim P., Janavs J., Weiller E., Dunbar G.C. (1998). The Mini-International Neuropsychiatric Interview (MINI): The development and validation of a structured diagnostic psychiatric interview for DSM-IV and ICD-10. J. Clin. Psychiatry.

[44] Montgomery S.A., Asberg M. (1979). A new depression scale designed to be sensitive to change. Br. J. Psychiatry.

[45] Young R.C., Biggs J.T., Ziegler V.E., Meyer D.A. (1978). A rating scale for mania: Reliability, validity and sensitivity. Br. J. Psychiatry.

[46] Lykouras L., Oulis P., Psarros K., Daskalopoulou E., Botsis A., Christodoulou G.N., Stefanis C. (2000). Five-factor model of schizophrenic psychopathology: How valid is it?. Eur. Arch. Psychiatry Clin. Neurosci..

[47] Kay S.R., Fiszbein A., Opler L.A. (1987). The Positive and Negative Syndrome Scale (PANSS) for Schizophrenia. Schizophr. Bull..

[48] Pfungst O. (1911). Clever Hans (The Horse of Mr. von Osten) a Contribution to Experimental Animal and Human Psychology.

[49] Johnston P.J., Devir H., Karayanidis F. (2006). Facial emotion processing in schizophrenia: No evidence for a deficit specific to negative emotions in a differential deficit design. Psychiatry Res..

[50] Johnston P.J., McCabe K., Schall U. (2003). Differential susceptibility to performance degradation across categories of facial emotion—A model confirmation. Biol. Psychol..

